# A Chromosome-level Genome Assembly of the Western Nose-Horned Viper (*Vipera ammodytes ammodytes*)

**DOI:** 10.1093/gbe/evaf210

**Published:** 2025-11-10

**Authors:** Wei-Qiao Rao, Esperanza Rivera-de-Torre, Lorenzo Seneci, Min-Hui Shi, Yao-Lei Zhang, Liang Lin, Tian-Ming Lan, Jože Pungerčar, Si-Qi Liu, Andreas H Laustsen

**Affiliations:** Department of Biotechnology and Biomedicine, Technical University of Denmark, Kongens Lyngby DK-2800, Denmark; Department of Mass Spectrometry, BGI Genomics Co., Ltd., Shenzhen, China; Department of Biotechnology and Biomedicine, Technical University of Denmark, Kongens Lyngby DK-2800, Denmark; Department of Biotechnology and Biomedicine, Technical University of Denmark, Kongens Lyngby DK-2800, Denmark; Adaptive Biotoxicology Lab, School of the Environment, The University of Queensland, St. Lucia, QLD 4067, Australia; BGI Research, Wuhan 430074, China; State Key Laboratory of Agricultural Genomics, Key Laboratory of Genomics, Ministry of Agriculture, BGI Research, Shenzhen 518083, China; College of Wildlife and Protected Areas, Northeast Forestry University, Harbin 150040, China; State Key Laboratory of Agricultural Genomics, Key Laboratory of Genomics, Ministry of Agriculture, BGI Research, Shenzhen 518083, China; Department of Mass Spectrometry, BGI Genomics Co., Ltd., Shenzhen, China; College of Wildlife and Protected Areas, Northeast Forestry University, Harbin 150040, China; Department of Molecular and Biomedical Sciences, Jožef Stefan Institute, Ljubljana SI-1000, Slovenia; Department of Mass Spectrometry, BGI Genomics Co., Ltd., Shenzhen, China; Department of Biotechnology and Biomedicine, Technical University of Denmark, Kongens Lyngby DK-2800, Denmark

**Keywords:** genomics, snake, PacBio Sequel, *Vipera ammodytes ammodytes*

## Abstract

We present a chromosome-level genome assembly of the western nose-horned viper (*Vipera ammodytes ammodytes*), the most medically important viper in Europe. Using PacBio Sequel and Illumina HiSeq X Ten sequencing, we generated ∼270 Gb of data, achieving ∼131× coverage of the genome. The final assembly spans 1.55 Gb with a contig N50 of 45.9 Mb and a scaffold N50 of 210 Mb, anchored into 18 pseudo-chromosomes. Completeness was supported by recovery of 97.1% of Vertebrata BUSCOs. A total of 20,775 protein-coding genes were predicted, of which 96.6% were functionally annotated. Repetitive sequences accounted for 53.75% of the genome, dominated by LINEs (41.87%) and LTRs (14.35%). We identified 112 venom-related genes across 15 families, with expansions in SVMPs, Snaclecs, sPLA₂s, SPIs, and SVSPs, together comprising 62.5% of the venom repertoire. Chemosensory genes were also expanded, including 448 olfactory receptors, 72 taste receptors, and 29 vomeronasal receptors. This assembly represents the most complete genome resource for a true viper to date and provides a key resource for investigating venom evolution, chemosensory adaptation, and comparative snake genomics.

Significance The western nose-horned viper (*Vipera ammodytes ammodytes*) is arguably the most venomous snake in Europe. Here, we report a chromosome-level genome assembly generated using PacBio and Illumina sequencing. The assembly demonstrates high completeness (97.1% BUSCO recovery) and extensive functional annotation (96.6% of 20,775 predicted protein genes) and reveals expansions in venom and chemosensory gene families. This resource establishes *V. ammodytes ammodytes* as a reference genome for viperid snakes and a valuable model for future studies of snake venom systems and ecological adaptation.

## Introduction

Snakes are a diverse reptilian lineage with more than 3,500 species worldwide, occupying nearly all terrestrial ecosystems except Antarctica (Wallach et al. 2014). Their evolutionary success is linked to specialized traits such as unique locomotory modes, chemosensory systems, and, in venomous taxa, complex venom apparatuses (Savitzky 1983; Krochmal et al. 2004; Brischoux and Shine 2011; Title et al. 2024; Biswas et al. 2025). Among these, venoms (complex mixtures of protein-based toxins) exemplify rapid adaptive molecular evolution driven by ecological pressures such as prey specialization and resistance (Jackson 2003; Barlow et al. 2009; Jackson et al. 2019; Lyons et al. 2020).

The western nose-horned viper (*Vipera ammodytes ammodytes*) is the most venomous snake in Europe and a key representative of the subfamily Viperinae (true vipers) within the Viperidae family (Leonardi et al. 2019; Paolino et al. 2020). Its venom contains neurotoxic phospholipases A₂, known as ammodytoxins, which are responsible for severe envenomation outcomes (Ritonja and Gubenšek 1985; Georgieva et al. 2008) and displays marked geographic and subspecific variation (Hempel et al. 2018; Lakušić et al. 2025; Talavera et al. 2025). Despite its biomedical importance, high-quality genomic resources for *V. ammodytes ammodytes* remain limited. Most available snake genomes are from elapids or pit vipers (subfamily Crotalinae), while assemblies for true vipers have been scarce and of lower contiguity (Rao et al. 2022).

In this study, we present a chromosome-level genome assembly and venom gland transcriptome of *V. ammodytes ammodytes*. This resource fills a phylogenetic gap within the Viperinae and enables detailed study of venom gene repertoires, chemosensory adaptation, and comparative snake genomics.

## Results and Discussion

### Genome Sequencing and Assembly

**Fig. 1.**
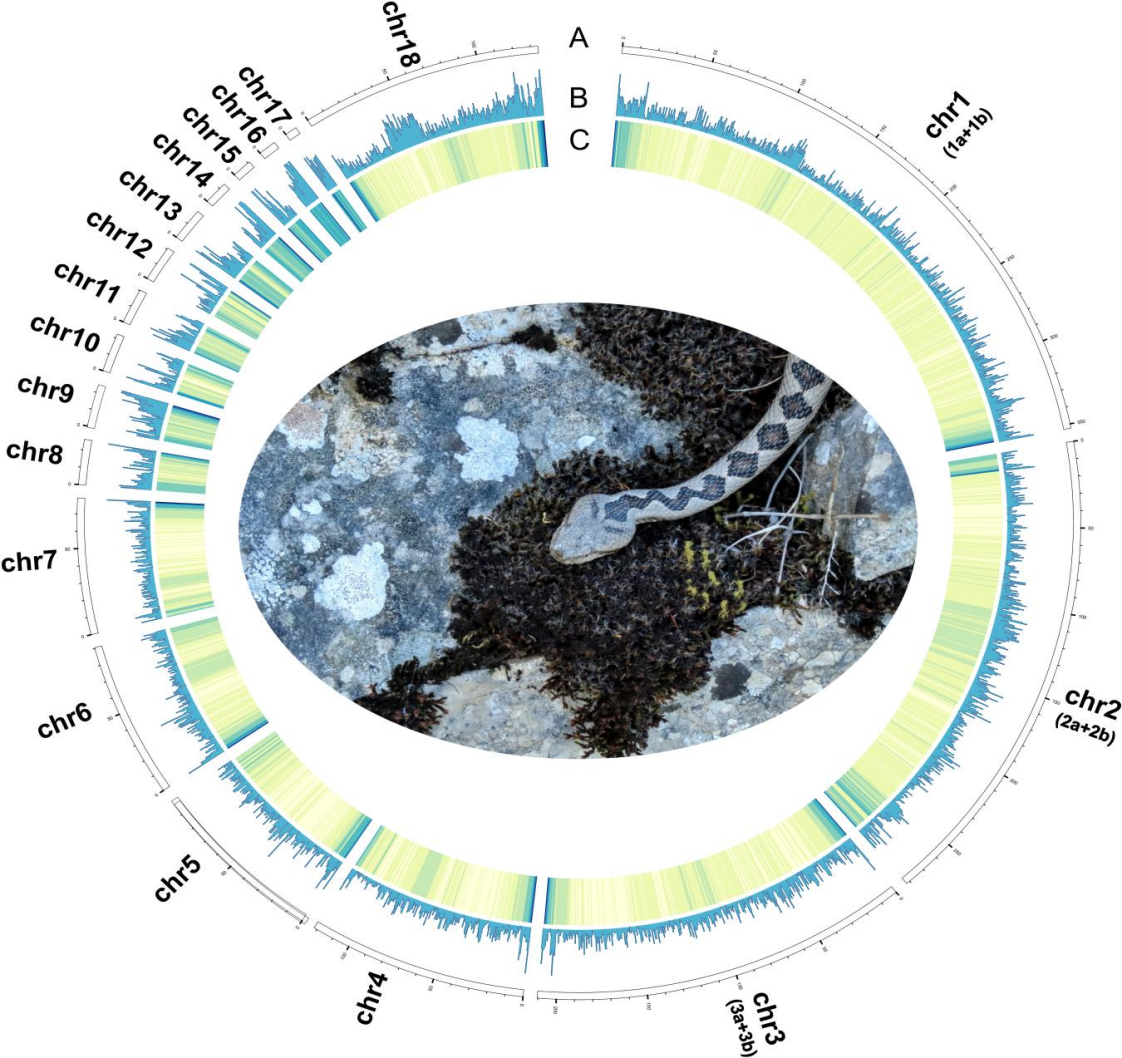
Circos plot of the *V. ammodytes ammodytes* genome assembly showing sizes (each single scale represents 15 Mb) of 18 pseudo-chromosomes, corresponding to a complete set of the presumed 21 chromosomes. Tracks (outer to inner circles) indicate the following: a) the chromosome length of *V. ammodytes ammodytes*, with units in Mb; b) gene density at 500 kb bins (gene numbers per Mb; minimum 0, maximum 100); c) GC content at 500 kb bins (the darker the color, the larger the proportion). The assembly based on the *V. latastei* genome is composed of 17 autosomes (7 macro-, 10 micro-) and a sexual chromosome (Chr 18, Chr Z).

Using PacBio Sequel long reads and Illumina HiSeq X Ten short reads, we generated ∼270 Gb of data, corresponding to ∼131× coverage (Table 1). Upon completing PacBio data correction, trimming, and assembly through NextDenovo (Hu et al. 2023), a draft genome was assembled, featuring 296 contigs with an overall size of 1,552 Mb and a contig N50 of 45.894 Mb. Following the refinement of the draft genome with PacBio and Illumina sequencing data, NextPolish (Hu et al. 2020) and purge_dups (Guan et al. 2020) were employed to eliminate accuracy and redundancy, resulting in a purged genome comprising 261 contigs, totaling 1,546 Mb, and a contig N50 of 45.90 Mb. The final assembly has a total span of 1.55 Gb with a contig N50 of 45.90 Mb and a scaffold N50 of 210 Mb and was anchored into 18 pseudo-chromosomes using the genome of the closely related *Vipera latastei* as a reference (Talavera et al. 2025) using RagTag (Alonge et al. 2022). These metrics compare favorably with other snake genomes, such as *Crotalus tigris* (Schield et al. 2019) and *Naja naja* (Suryamohan et al. 2020), and represent the highest-quality assembly available for a true viper (Fig. 1).

**Table 1:** Genome assembly statistics and comparison with other snake genomes

…	*V. ammodytes ammodytes*	*V. latastei*	*C. tigris*	*B. jararaca*	*A. feae*	*N. naja*	*H. major*
Assembly size, Gb	1.55	1.63	1.61/1.59	2.10	1.56	1.79	2.17
Number of scaffolds	24	56	380	27,698	…	1,897	1,320
Scaffold N50, Mb	210	220	207.72	…	…	223.35	268
Number of contigs	261	N/A	4,228	28,752	4,303	13,066	1,815
Contig N50, Mb	45.90	44.84	2.11	0.16	1.59	0.300	…
GC content, %	40.7	40.96	39.9	40.5	…	40.5	…
Venom protein-coding genes	112	31	51	86	…	139	…
Anchored chromosomes	18	18	18	N/A	…	19	16
Assembly BUSCO completeness, %	97.1	97.1	95.8	92.30	92.40	94.30	96.0
Assembly BUSCO fragmented, %	0.9	…	2	9.6	2.7	6.7	0.8
Annotation BUSCO completeness, %	93.3	98.6	-	-	-	-	-
Reference	This study	Talavera et al. (2025)	Margres et al. (2021)	Almeida et al. (2021)	Myers et al. (2022)	Suryamohan et al. (2020)	Ludington et al. (2023)

### Assembly Completeness and Annotation

We evaluated the completeness of the *V. ammodytes ammodytes* genome using BUSCO (Benchmarking Universal Single-Copy Orthologs) datasets (Simão et al. 2015). From 3,354 ortholog groups searched in BUSCO (vertebrata_odb10), 3,257 (97.1%) of expected orthologs were recovered (96.3% single-copy, 0.8% duplicated), and 29 (0.9%) genes were “fragmented.” A total of 20,674 protein-coding genes were predicted, of which 96.6% could be functionally annotated using SwissProt, TrEMBL, KEGG, InterPro, and GO databases (Table S1). Repetitive elements accounted for 53.75% of the genome, dominated by LINEs (41.87%) and LTRs (14.35%), consistent with patterns in other viperid genomes (Rao et al. 2022; Peng et al. 2023). Some of the repetitive sequences were classified within two or more categories.

### Comparative Genomics

Comparative analyses across seven snake genomes revealed conserved synteny between *V. ammodytes ammodytes* and *V. latastei*, with similar genome sizes (1.55 Gb vs. 1.63 Gb) and scaffold N50s (210 Mb vs. 222 Mb; Table 1). Repeat content in *V. ammodytes ammodytes* was notably higher than in some elapids such as *N. naja* (10.0% LINEs, 8.2% LTRs) (Suryamohan et al. 2020) and *Bungarus multicinctus* (18.7% LINEs, 3.76% LTRs) (Liu et al. 2023), although in the same 55% to 60% total repeat range as several sea snakes (*Hydrophis* spp.) (Li et al. 2021; Ludington et al. 2023).

### Venom Gene Repertoire

We identified 112 venom-related genes across 15 protein families (Fig. S1). The five dominant families [snake venom metalloproteases {SVMPs, *n* = 20}, snake C-type lectins {Snaclecs, *n* = 19}, secreted phospholipases A₂ {sPLA₂s, *n* = 11}, serine protease inhibitors {SPIs, *n* = 10}, and snake venom serine proteases {SVSPs, *n* = 10}] accounted for 62.5% of the venom repertoire. Tandem duplications were especially evident in SVMP and sPLA₂ clusters, consistent with the ongoing diversification of toxin families (Fig. S2) (Fry 2005; Giorgianni et al. 2020). The transcriptome data confirmed that the expanded toxin families (SVMPs, Snaclecs, sPLA_2_s, SPIs, and SVSPs) are highly expressed, consistent with proteo-transcriptomic studies of *V. ammodytes ammodytes* venom (Leonardi et al. 2015; Hempel et al. 2018). This concordance suggests that the genomic distribution of these venom gene families directly reflects their dominant role in the expressed venom proteome. Moreover, the abundance of LINEs and other repeats (∼54% of the genome) further suggests that transposable elements may facilitate rapid genomic innovation particularly within toxin gene clusters (Lyons et al. 2020; Margres et al. 2021).

### Genomic Basis of Sensory Perception

Comparative genomics revealed a reduced vomeronasal receptor (V2R) repertoire in *V. ammodytes ammodytes* (73 genes) relative to pit vipers such as *C. tigris* (374) and elapids like *N. naja* (203), suggesting lineage-specific contractions linked to sensory trade-offs. Olfactory receptor (OR) copy numbers were likewise lower than in visually oriented foragers such as *N. naja* and *Thermophis baileyi* and markedly below the expanded repertoires of pythons (*Python bivittatus*) and other colubrids (Fig. S3) (Biswas et al. 2025). These patterns indicate repeated, clade-specific episodes of receptor expansion and contraction across snakes, highlighting the importance of chemoreception in ecological adaptation. Notably, the high abundance of LINEs and other repeats in the *V. ammodytes ammodytes* genome may further contribute to this dynamic by facilitating rapid genomic innovation within sensory receptor gene clusters.

## Conclusion

We present a chromosome-level genome assembly for the western nose-horned viper (*V. ammodytes ammodytes*), generated using PacBio and Illumina sequencing. The assembly spans 1.55 Gb with a scaffold N50 of 210 Mb and a contig N50 of 45.9 Mb, anchored into 18 pseudo-chromosomes. BUSCO analysis confirmed high completeness (97.1%), and functional annotation assigned roles to 96.6% of the 20,775 predicted protein-coding genes. This resource represents the most complete genome available for a true viper and provides a foundation for comparative genomics, venom biology, and translational research in toxinology and antivenom development.

## Materials and Methods

### Sample Collection

With permission from the Slovenian Ministry of Environment and Spatial Planning (permit number 35601-32/2018-5), an adult male *V. ammodytes ammodytes* was collected in the wild near Jesenice, in the northwestern region of Slovenia. The specimen was maintained for several days in a large cage with access to water until venom extraction, in full compliance with Directive 2010/63/EU of the European Parliament and Council on the protection of animals used for scientific purposes. Two days after venom extraction, the snake was humanely euthanized by captive bolt, followed by pithing of the brain to ensure loss of consciousness. Dissection was performed immediately, and liver and venom gland tissues were flash-frozen in liquid nitrogen and stored at −80 °C until use.

### DNA Extraction

High-molecular-mass genomic DNA (>100 kb) was extracted from frozen liver tissue of *V. ammodytes ammodytes* using the MegaLong Purification Kit (G-Biosciences, Saint Louis, United States) following a modified protocol. Briefly, deeply frozen liver tissue was ground in liquid nitrogen, and cell nuclei were isolated according to the manufacturer's instructions. The nuclei were digested with proteinase K, after which dialysis was performed overnight using Spectra/Por 2 tubing (12 to 14 kDa cutoff; Spectrum Laboratories, San Francisco, United States) against 10 mM Tris-HCl (pH 8.0) and 1 mM EDTA, with three buffer changes. The resulting genomic DNA was stored at 4 °C, and concentration was determined using both a NanoDrop spectrophotometer (Thermo Fisher Scientific) and a Qubit 2.0 fluorometer (Invitrogen).

### Whole-Genome Sequencing

Whole-genome sequencing for de novo assembly was performed at the BGI–Wuhan Sequencing Center. PacBio Sequel single-molecule real-time (SMRT) sequencing (18 cells, Sequencing Chemistry 2.0 kit; RRID:SCR_017989) generated 19.7 million reads with a total yield of 199 Gb. In parallel, Illumina HiSeq X Ten sequencing (RRID:SCR_016385) produced 150 bp paired-end (PE150) reads. Paired-end libraries with insert sizes of 410 and 670 bp, as well as mate-pair libraries with insert sizes of 2, 5, 10, and 20 kb, were constructed following Illumina's standard protocol. When combined, the datasets provided approximately 131× coverage of the *V. ammodytes ammodytes* genome.

### RNA Extraction and Transcriptomics

Total RNA was extracted from the venom gland of *V. ammodytes ammodytes* using TRIzol reagent (Invitrogen). RNA quality and quantity were assessed with an Agilent 4200 Bioanalyzer and RNA 6000 Nano LabChip Kit (Agilent). One microgram of poly(A)-enriched RNA was used to prepare libraries with the MGIEasy RNA Directional Library Prep Kit (MGI Tech). Libraries were sequenced on the MGISEQ-2000 platform according to the manufacturer's instructions, generating 150 bp paired-end reads (PE150).

### Genome Assembly

Draft genomes of *V. ammodytes ammodytes* were assembled from PacBio long reads using NextDenovo v2.5.0 (Hu et al. 2023). Assembly polishing was performed with NextPolish v1.4.0 (Hu et al. 2020), using two rounds of Illumina short-read data to improve base accuracy. Redundant sequences were removed with purge_dups v1.2.5 (Guan et al. 2020), and scaffolding produced a de novo genome assembly at the scaffold level. We utilized NCBI FCS-GX (Astashyn et al. 2024) to screen the assembled genome for foreign contamination, and no such contamination was detected; all contigs were consistent with the expected organism classification. Finally, we used RagTag (Alonge et al. 2022) to scaffold our de novo assembly to the reference genome of *V. latastei* genome assembly (Talavera et al. 2025). Default parameters were applied unless otherwise specified. Assembly completeness was evaluated with BUSCO v5.4.0 (Manni et al. 2021) using the vertebrata_odb10 dataset. The final genome assembly has been deposited in NCBI under BioProject accession PRJNA1177245.

### Repeat Annotation

Transposable elements (TEs) and other repetitive sequences in *V. ammodytes ammodytes* were identified using a combination of homology-based and de novo approaches. For the homology-based approach, the genome assembly was aligned to REPBASE v21.01 using RepeatMasker v4.0.5 (Chen 2004), RepeatProteinMask, and Tandem Repeats Finder v4.07b (Ou and Jiang 2018). For the de novo approach, RepeatModeler v2.0 (Flynn et al. 2020) and LTR_retriever were used to construct a custom repeat library. In total, 53.75% of the *V. ammodytes ammodytes* genome consisted of repetitive elements, with LINEs representing the predominant class (∼42% of the genome). All identified repeats were masked prior to gene annotation.

### Gene Annotation

Protein-coding genes were predicted using a combination of homology-based, de novo, and transcriptome-based approaches. For the homology-based method, GeneWise v2.4.1 (Birney et al. 2004) was used to align protein sequences from six related snake species (*V. latastei*, *C. tigris*, *Bothrops jararaca*, *Azemiops feae*, *N. naja*, and *H. major*) downloaded from NCBI, with an *E*-value cutoff of 1e^−5^. For the de novo method, the repeat-masked genome was analyzed with AUGUSTUS v3.0.3 (Stanke et al. 2004). For the transcriptome-based method, transcripts were assembled from RNA-seq data using StringTie v1.3.3b (Pertea et al. 2015). The MAKER pipeline v3.01.03 (Campbell et al. 2014) integrated the results of all three approaches to produce the final gene set. In total, 20,775 protein-coding genes were annotated in the *V. ammodytes ammodytes* genome.

### Functional Annotation

Functional annotation of predicted protein-coding genes was performed using BLAST (*E*-value cutoff 1e^−5^) against SwissProt, TrEMBL, KEGG, InterPro, and Gene Ontology (GO) databases. InterProScan v5.52-86.0 (Jones et al. 2014) was used to predict domains and motifs. In total, 96.6% of the 20,775 predicted protein-coding genes were functionally annotated across these resources. Noncoding RNAs, including tRNAs, rRNAs, snRNAs, and miRNAs, were also predicted: tRNAs were identified with tRNAscan-SE v1.3.1 (Lowe and Eddy 1997), and other ncRNAs were annotated by comparison with the Rfam database (Release 12.0). BLASTP was used to compare the identified functional genes (olfactory receptor genes and venom genes) against the NCBI nonredundant protein database to ensure no false annotations were present.

## Supplementary Material

evaf210_Supplementary_Data

## Data Availability

The raw sequencing data and assembled genomes that support the findings of this study have been deposited into the CNGB Sequence Archive (CNSA) (Guo et al. 2020) of the China National GenBank Database (CNGBdb) (Chen et al. 2020), https://db.cngb.org/cnsa/ with accession number CNP0002260, and into NCBI with the BioProject ID PRJNA1177245. This paper does not report the original code of the bioinformatic analysis.
