# Supplementary material for: A Chromosome-level Genome Assembly of the Western Nose-Horned Viper (Vipera ammodytes ammodytes)
Source: Genome Biol Evol. 2025 Nov 10;17(12):evaf210. doi: 10.1093/gbe/evaf210 (PMC12680307; doi:10.1093/gbe/evaf210)
Supplement: evaf210_Supplementary_Data [file evaf210_supplementary_data.docx]

*Genome Resource*

**A chromosome-level genome assembly of the western nose-horned viper (*Vipera ammodytes ammodytes*)**

Wei-qiao Rao^1,2#^, Esperanza Rivera-de-Torre^1#^, Lorenzo Seneci^1,3^, Min-hui Shi^4,5,6^, Yao-lei Zhang^5^, Liang Lin^2^, Tian-ming Lan^6^, Jože Pungerčar^7*^, Si-qi Liu^2*^, Andreas H. Laustsen^1*^

^1^Department of Biotechnology and Biomedicine, Technical University of Denmark, DK-2800 Kongens Lyngby, Denmark

^2^Department of Mass Spectrometry, BGI Genomics Co., Ltd., Shenzhen, China

^3^Adaptive Biotoxicology Lab, School of the Environment, The University of Queensland, 4067 St. Lucia, QLD, Australia

^4^BGI Research, Wuhan 430074, China

^5^State Key Laboratory of Agricultural Genomics, Key Laboratory of Genomics, Ministry of Agriculture, BGl Research, Shenzhen 518083, China

^6^College of Wildlife and Protected Area, Northeast Forestry University, Harbin 150040, China

^7^Department of Molecular and Biomedical Sciences, Jožef Stefan Institute, SI-1000 Ljubljana, Slovenia

# Both authors have contributed equally

*Corresponding authors:

Jože Pungerčar; joze.pungercar@ijs.si; Tel.: +386-1-477-3713

Si-qi Liu; siqiliu@genomics.cn; Tel.: +86-1391-1002-1096

Andreas Hougaard Laustsen; [ahola@bio.dtu.dk](mailto:ahola@bio.dtu.dk); Tel.: +45-2988-1134

**Supplementary table 1.** Repetitive content and function annotation of predicted genes statistics for *V. ammodytes*

| **Repetitive content** | | | |
| --- | --- | --- | --- |
|  | Total repetitive elements | | 53.75% |
|  | DNA transposons | | 4.29% |
|  | Long Interspersed Nuclear Elements (LINEs) | | 41.87% |
|  | Short Interspersed Nuclear Elements (SINEs) | | 1.93% |
|  | Long Terminal Repeats (LTR) | | 14.35% |
|  | Other | | 0.00% |
|  | Unclassified repeats | | 0.23% |
| **Gene functional annotation** | | | |
|  | Total predicted protein-coding genes | 20,674 | |
|  | Swiss-Prot | 16,987 | |
|  | KEGG | 15,950 | |
|  | InterPro | 18,685 | |
|  | TrEMBL | 19,431 | |
|  | GO | 12,940 | |


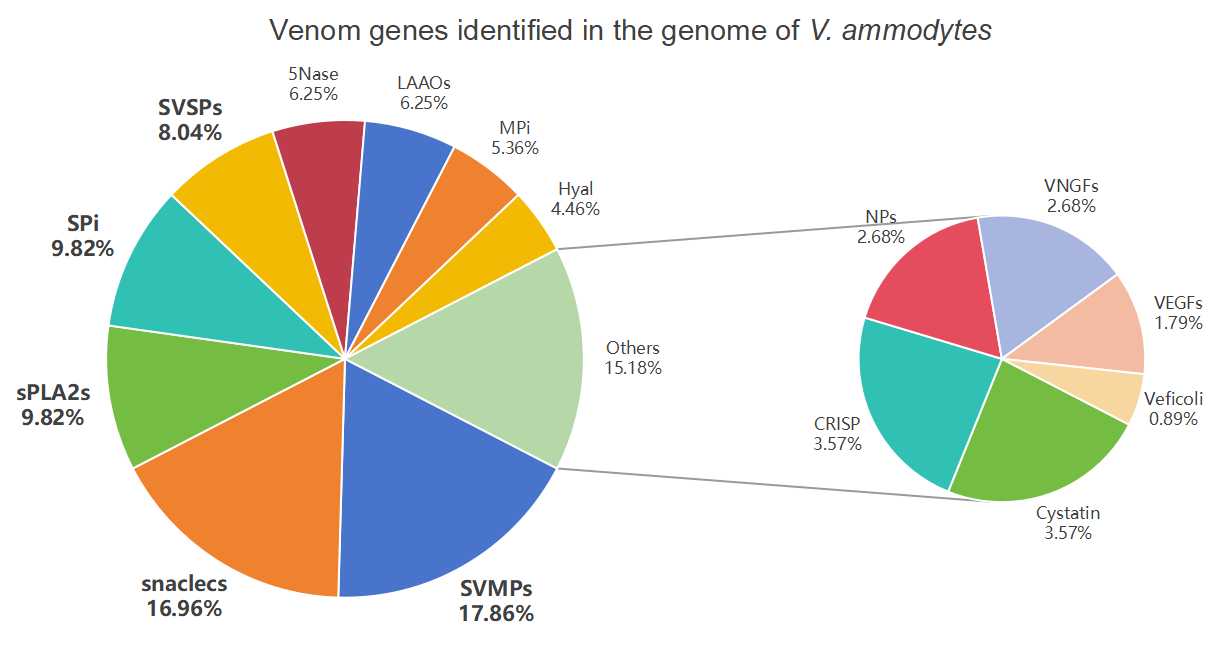


**Supplementary Figure 1.** Venom gene family distribution of *V. ammodytes ammodytes*. A total of 112 venom-encoding genes, which are categorized into 15 distinct protein families, have been identified within its genome. Among these, five major toxin families (SVMPs, Snaclec, sPLA_2_s, SPi, and SVSPs) are highlighted in bold in the pie chart figure and collectively account for 62.5% (over 60%) of all toxin-encoding genes in the venom of *V. ammodytes ammodytes*.

**
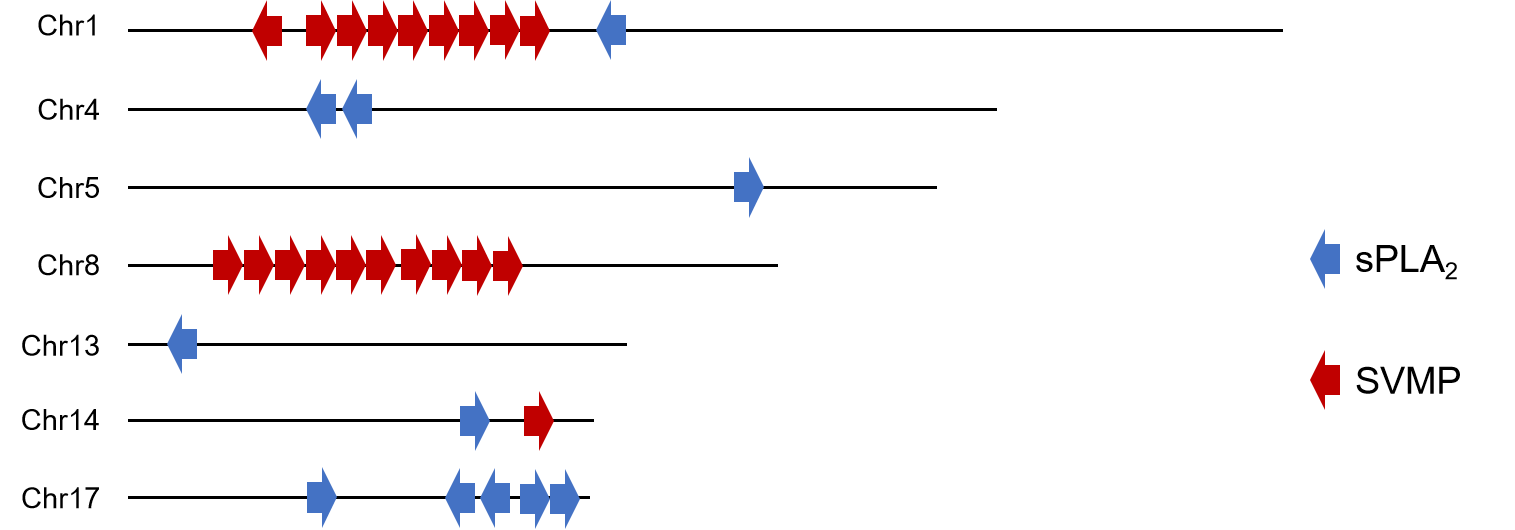
**

**Supplementary Figure 2**. Syntenic regions of the *V. ammodytes* *ammodytes* containing sPLA_2_ and SVMP genes.


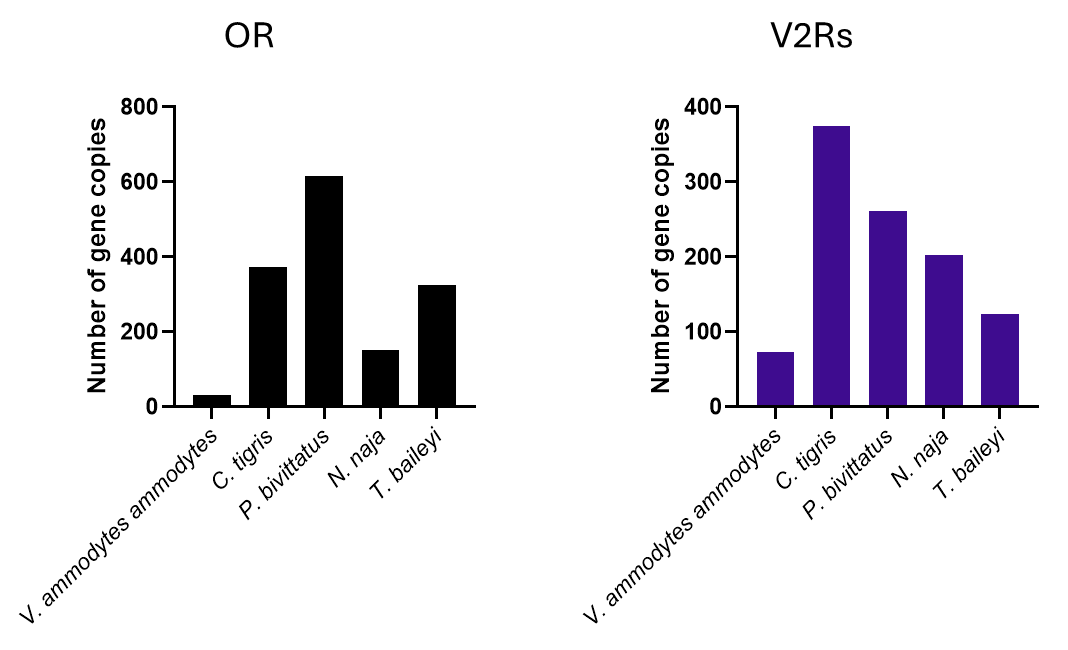


**Supplementary Figure 3**. Number of gene copies for olfactory receptors (ORs) and thermal sensing channels (V2Rs) for V. ammodytes amodytes, C. tigris, P. bivittatus, and T. baileyi.
